# Sternalis Muscle: An Unexpected Finding during Mastectomy

**DOI:** 10.1155/2015/723198

**Published:** 2015-11-02

**Authors:** Prakash K. Sasmal, Susanta Meher, Tushar S. Mishra, N. Deep, Prabhas R. Tripathy, Satyajit Rath

**Affiliations:** ^1^Department of Surgical Discipline, All India Institute of Medical Sciences, Bhubaneswar 751019, India; ^2^Department of Radiodiagnosis, All India Institute of Medical Sciences, Bhubaneswar 751019, India; ^3^Department of Anatomy, All India Institute of Medical Sciences, Bhubaneswar 751019, India

## Abstract

Sternalis muscle also called rectus sternalis, rectus thoracis, or episternalis is an anomalous muscle of the anterior chest wall with unknown anatomical function. It is regularly observed in lower animal but infrequently in humans. Presence of this muscle can create confusion with tumours of the anterior chest wall during routine mammography. Although less is known about its origin and innervations, knowledge about this muscle can have many clinical implications. A case of unilateral sternalis muscle detected during mastectomy, in a female with carcinoma of the right breast, is being reported with a brief review of the literature and highlighting its clinical significance.

## 1. Introduction

Sternalis muscle is an uncommon anatomical variant of anterior chest wall muscles [1]. It is a vertical strip of thin ribbonlike muscle located in the parasternal region, superficial to pectoralis major, with its fibre oriented parallel to the sternum and perpendicular to the fibres of the pectoralis major muscle. Cabrollius, in 1604, was the first to report the presence of this entity, although Du Puy, in 1726, gave a more precise description of it [2, 3]. Various nomenclatures of this muscle are found in the literature including musculus sternalis presternalis, sternalis muscle, sternalis brottrum, or thoracis [4, 5]. This report of an incidentally detected sternalis muscle is being presented for its clinical significance in day-to-day clinical practice.

## 2. Case Report

**Figure 1:**
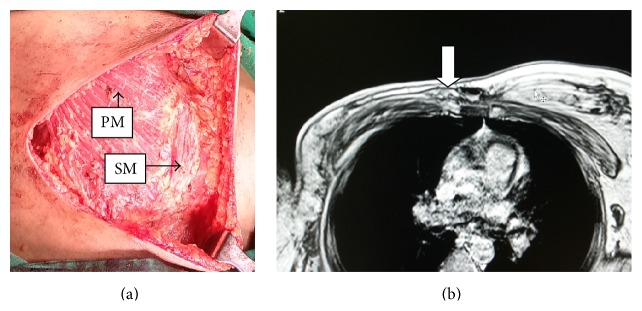
(a) Intraoperative photograph showing a thin fleshy ribbonlike muscle of size approximately 10 cm × 3 cm present in the right parasternal region perpendicular to fibres of the pectoralis major muscle. PM: pectoralis major, SM: sternalis muscle. (b) MRI of anterior chest wall (post-MRM) showing a unilateral sternalis muscle (white arrow) in transverse section.

A 39-year-old female patient presented to the Surgical Outpatient Department of AIIMS, Bhubaneswar, with a right breast lump of approximately 3 cm in diameter. On evaluation she was found to have a T_2_N_1_M_0_ carcinoma in her right breast. After a detailed workup she was posted for modified radical mastectomy of the right breast. During mastectomy a thin ribbonlike muscle was found in the parasternal area with its fibres oriented craniocaudally, parallel to the sternum and perpendicular to the fibres of the right pectoralis major muscle (Figure 1(a)). The muscle was thin, approximately 10 cm in length and 3 cm in breadth. Fibrofatty breast tissue was found below the muscle which was cleared during dissection. It was arising from the sternum below the sternal head of the sternocleidomastoid muscle of the right side and inserted into the costal cartilages of the 5th and 6th right rib and its tendon was separate from the rectus abdominis muscle. Magnetic resonance imaging (MRI) of the anterior chest wall was done postoperatively after taking due consent from the patient, to look for any similar muscle on the opposite site (Figure 1(b)) although it was found to be absent.

## 3. Discussion

In 2001, Jelev et al. defined the characteristics of the muscle as (1) location between the anterior thoracic fascia and pectoral fascia, (2) origin from the sternum or infraclavicular area, and (3) its insertion into the rectus sheath, lower ribs, costal cartilages, or external oblique aponeurosis [1, 6]. In our case the muscle fits into the above-mentioned criteria of sternalis muscle. In most cases it is unilateral with an equal incidence in males and females; however the frequency of occurrence varies among various ethnic groups [3]. The highest incidence is seen among Asians (11.5%) whereas the reported incidence in India is around 4–8% [7]. A higher incidence (48%) of this muscle has been found in association with anencephaly [8].

Since its discovery different theories have been proposed to explain its embryological origin although the consensus is still lacking. Most of the authors support its origin from one among the adjacent muscles such as pectoralis major, rectus abdominis, sternocleidomastoid, or panniculus carnosus [1]. According to Sadler [4] and Saeed et al. [9], it is a part of the ventral longitudinal muscle column arising from the ventral lips of hypomere which is represented by rectus abdominis muscle in abdomen and strap muscles in the neck and in the thorax in which longitudinal muscle disappears but occasionally represented by rectus sternalis [6, 8]. According to Barlow [10], it represents remains of panniculus carnosus, whereas Saeed et al. [9] suggest that it could be arising from pectoralis major with innervations from pectoral nerve or from rectus abdominis with innervations from intercostal nerves [6, 8]. The sternalis muscle takes its nerve supply from the internal or external thoracic or pectoral nerve in 55% of cases, intercostal nerves in 43% of cases, and both in 2% of cases [1, 5]. Kida and Kudoh found the sternalis muscle to be supplied by the pectoral nerves. Branches of intercostal nerve may pierce the muscle but do not directly supply the muscle [11]. The arterial supply of this muscle is from perforating branches of internal thoracic artery. It has been suggested that contraction of this muscle can elevate the lower part of the chest because of its particular location. Thus it plays only an accessory role in lower chest wall elevation [1, 12].

*Clinical Significance*. Although it has been well described in the literature and well known to trained anatomist, knowledge among physicians, surgeons, oncologists, and radiotherapists dealing with the diseases of anterior chest wall is deficient [8]. Bailey et al. found a near-total unfamiliarity of this muscle in their survey among physicians, medical students, surgeons, and faculty of other disciplines [13]. This is because in most of the standard anatomical textbooks it is not adequately mentioned. With the advent of more sophisticated diagnostic tools and therapeutic modalities the importance of this muscle has been reemphasized. Renewed interest about this muscle among clinicians is because of the following reasons.

1. During routine mammography sternalis muscles can be mistaken for a tumour in the craniocaudal view during initial investigation or as a recurrence during follow-up in the postoperative period. Presence of this muscle can be confirmed by computed tomography (CT) or magnetic resonance imaging (MRI) and craniocaudal view in mammography [1, 6].
2. It may be confused with hernia of the major pectoralis muscle by the examining clinician [12].
3. During radiotherapy the depth at which internal thoracic nodes are irradiated may vary in presence of this muscle [14].
4. Presence of sternalis muscle can cause changes in electrical activities during electrocardiography [1, 8].
5. It may interfere with submuscular pocket dissection when a submammary approach is used during augmentation mammoplasty [12].
6. During oncological procedures it is important to excise this muscle as part of breast tissue lies deep in it [8, 15].
7. It can also be used to cover prosthesis in the most medial part during augmentation mammoplasty [8, 16].
8. If detected preoperatively sternalis muscle can be used in reconstructive surgery [1].

## 4. Conclusion

The last few decades have witnessed a lot of advances in medical science in the form of new diagnostic and therapeutic modalities. Because of this there is high probability that sternalis muscle will be detected more frequently than before. Although well known to anatomist it is relatively unfamiliar among surgeons and radiologists. Presence of this muscle can cause diagnostic dilemma which can be confirmed by CT or MRI scan. If detected preoperatively it can be used in various reconstructive procedures during surgery. When detected intraoperatively during mastectomy for carcinoma of the breast, it should ideally be removed for complete clearance of the breast tissue.
